# Seroprevalence and determinants of toxoplasmosis in pregnant women attending antenatal clinic at the university teaching hospital, Lusaka, Zambia

**DOI:** 10.1186/s12879-016-2133-7

**Published:** 2017-01-05

**Authors:** Christiana Frimpong, Mpundu Makasa, Lungowe Sitali, Charles Michelo

**Affiliations:** 1 Department of Public Health, University of Zambia, School of Medicine, Lusaka, Zambia; 2 Department of Biomedical Science, University of Zambia, School of Medicine, Lusaka, Zambia

**Keywords:** Determinants, Pregnant women, Seroprevalence, Toxoplasmosis, University teaching hospital (UTH), Zambia

## Abstract

**Background:**

Toxoplasmosis is a neglected zoonotic disease which is prevalent among pregnant women especially in Africa. This study aimed to determine the seroprevalence and determinants of the disease among pregnant women attending antenatal clinic at the University Teaching Hospital (UTH).

**Method:**

A cross-sectional study was employed where 411 pregnant women attending antenatal clinic at UTH were interviewed using closed ended questionnaires. Their blood was also tested for *Toxoplasma gondii* IgG and IgM antibodies using the OnSite Toxo IgG/IgM Combo Rapid test cassettes by CTK Biotech, Inc, USA.

**Result:**

The overall seroprevalence of the infection (IgG) was 5.87%. There was no seropositive IgM result. Contact with cats showed 7.81 times the risk of contracting the infection in the pregnant women and being a farmer/being involved in construction work showed 15.5 times likelihood of contracting the infection. Socio-economic status of the pregnant women also presented an inverse relationship (showed association) with the infection graphically. However, though there were indications of association between contact with cats, employment type as well as socioeconomic status of the pregnant women with the infection, there was not enough evidence to suggest these factors as significant determining factors of *Toxoplasma gondii* infection in our study population.

**Conclusion:**

There is a low prevalence of *Toxoplasma gondii* infection among pregnant women in Lusaka, Zambia. Screening for the infection among pregnant women can be done once or twice during pregnancy to help protect both mother and child from the disease. Health promotion among women of child bearing age on the subject is of immense importance in order to help curb the situation. Further studies especially that of case–control and cohort studies should be carried out in the country in order to better ascertain the extent of the condition nationwide.

## Background

Toxoplasmosis is a neglected zoonotic disease caused by a blood protozoan parasite called *Toxoplasma gondii* [1–4]. The organism is found worldwide and it infects nearly all warm-blooded animals including human beings [5, 6]. Once human beings contract the infection, they remain infected for life [7] hence, transmitting the disease vertically to their unborn babies [8].

The life cycle of *T. gondii* constitutes the sexual reproduction component in both wild and domestic cats (definitive hosts) and the asexual component in all warm-blooded animals including human beings (intermediate hosts) [9, 10] who get infected directly through handling contaminated cat litter boxes, meat and vegetables [8], blood transfusion [11] and organ transplants [7, 10, 11] or indirectly through eating improperly cooked contaminated meat or vegetables [8, 10]. Once in the human being, the organism lodges in the muscle tissues and then into the womb to infect the fetus; in the case of a pregnant woman [10, 11]. *Toxoplasma gondii* is found commonly in the tissues of pigs, sheep and goats [8, 10, 12] with prevalence of the disease ranging from 2.1-68% in swine, sows, cats, rats and mice [12] and 39% in pigs, 26.8% in goats and 33.2% in sheep [13].

A third of the global human population is believed to have had exposure with the organism and may have chronic infections [14]. Most people who get infected remain asymptomatic until their immune system is weakened, which paves way for clinical conditions to set in [15]. The infection is said to range from being asymptomatic to overt disease and can actually cause outbreaks [7, 13, 16]. Toxoplasmosis is particularly grave in pregnant women who get infected during gestation, congenitally infected fetuses and newborns, immunocompromised patients, and people with chorioretinitis [17]. In pregnant women, vertical transmission of the infection during the first trimester is critical and causes severe clinical conditions in the fetus, whereas third trimester infections have rapid transmission rate of parasites to fetuses, hence causing higher incidence of disease in the baby [11, 16]. Some general clinical manifestations of the infection are ocular disease, lymphadenopathy (most common), encephalitis and generalized infection in immunocompromised people [15, 18]. It causes spontaneous abortion of fetuses as well as stillbirths [16]. Surviving babies on the other hand develop neurological diseases such as epileptic seizures, choroidoretinitis, hydrocephalus, intra-cerebral calcification, mental retardation and deafness at a stage in their lifetime [11, 16, 17].

Toxoplasmosis in pregnant women varies geographically. There are reports of 3.7% in Korea [19], 6.4% in South Africa [20], 17.3% in London [21], 24.1% in Saudi Arabia [19], 28.3% in Thailand [22], 30.9% in Tanzania [23], 68.6% in Brazil [24] and 92.5% in Ghana [13].

Some factors associated with toxoplasmosis in pregnancy include, eating undercooked or cured meat (meat with preservatives such as salt, nitrates or sugar added) [25–27], having a pet cat [25, 27], contact with soil [26], educational level and occupation [28, 29], age and crowded conditions [28], being foreign born / race [28, 30], Parity [30] and eating raw vegetables [27].

Screening is very vital in detecting this infection [18, 31], as it has proven to be in France and Austria through the implementation of routine monthly screening of pregnant women for the disease. This has as well provided enough serological data on the disease in these two countries [11, 32].

The exact burden of Toxoplasmosis in pregnancy in Zambia is unknown and this is so because screening for *Toxoplasma gondii* in pregnant women is not routinely done in the country. Meanwhile, the Burden of the disease is reported to be generally severe in Immune compromised populations such as pregnant women and HIV patients [18]. This makes it difficult to detect and protect unborn babies from transplacental infections which bring about congenital Toxoplasmosis. As a result of this, babies, infants and children may suffer from disease conditions and complications and even death, which on the other hand could have been prevented. Compounding the situation further has been the lack of information on the determinants of the disease here in the country.

This study asks the question; ‘what is the prevalence of and factors associated with Toxoplasmosis among pregnant women attending antenatal clinic at the University Teaching Hospital (UTH) in Lusaka, Zambia? And therefore aimed to determine the prevalence and explore these factors.

In addition to providing the prevalence of *Toxoplasma gondii* infection in the referral hospital and to shed light on possible determining factors of the disease in the country, this study also set out to set the foundation for further studies to be carried out in this area. These will further create avenue for management and treatment of this disease in this population.

## Methods

### Study design

This was a cross-sectional study carried out at the national referral hospital - University Teaching Hospital (UTH), (15.4323° S, 28.3137° E), in Lusaka, the capital city of Zambia, from August to October 2015. The study consisted of all women at all stages of pregnancy. Women who did not consent to participate were not included in the study. This study employed estimation for simple proportion to calculate the sample size. The total sample size was 411, with a response rate of 80% using a prevalence of 31% from Tanzania, according to Mwambe et al. The first participant was randomly picked, after which every third consenting pregnant woman was interviewed and tested for the infection. There were 84 women in their first trimester, 134 in their second trimester and 192 in their third trimester. Of the 411 participants, 68 were HIV positive, 338 were HIV negative and 5 statuses were unknown.

Structured interviews with structured closed-ended questionnaires were used. The questionnaire was developed from information reviewed in literature. It gathered information on socio-demographic factors (Age, marital status, level of education, income; which is the money that comes in/a person makes in a month etc.), behavioral (‘eating meat’; which means eating meat and meat products, ‘length of cooking meat’; which is the number of minutes meat is cooked, ‘contact with cats’ which is ownership/a level of handling cats or cat liter, drinking unpasteurized milk is drinking raw fresh milk from the cattle), obstetrical (Parity is the number of times a woman has given birth, gestational age which is the stage of pregnancy in trimesters) and co-infection (HIV status) of the women. The women were not tested for HIV in this study but rather their HIV status was obtained with consent from their patient files. In addition to the questionnaire, blood samples were obtained for blood analysis for *T. gondii* infection. About 2mls of blood from the blood drawn from the women by the hospital for routine blood tests, was taken and used for the testing. For the pregnant women who were not undergoing routine blood testing by the hospital, about 2mls of venous blood was drawn from them as well and tested for the *Toxoplasma* infection. The OnSite Toxo IgG/IgM Combo Rapid test cassettes (a lateral flow chromatographic immunoassay) manufactured by the CTK Biotech Inc., San Diego, USA, were used in the laboratory to detect the presence of the anti-*Toxoplasma* antibodies in the blood samples, following manufacturer’s instructions.

### Data analysis

The data was analysed using STATA version 12 (StataCorp, Texas, USA). The quantile-quantile (Q-Q) plot was used to check normality of the continuous variable ‘age’ before it was categorized. The data was normally distributed hence, mean age and standard deviation was recorded. Chi-square test was used to determine associations between the infection and characteristics of the pregnant women through cross-tabulations, but where appropriate, the Fishers exact test was used instead of Chi-square test. Generalized linear model for binary outcome (in this case Toxoplasma infection present or absent), reporting odds ratios was used for univariate analysis. Significant variables obtained from univariate analysis together with priori for the study was put in the final model for multivariable analysis [20]. The corresponding odds ratios, confidence intervals and *p*-values (*p* < 0.05 was considered statistically significant) were recorded at 95% confidence interval. A new variable ‘socio-economic status’, was created out of all variables (income, residence, employment type and education) indicative of socio-economic status in our study and plotted against Toxoplasma infection in a graph.

## Results

Data from 411 pregnant women were obtained and analysed for this study. Most of the pregnant women (59.6%) were in the age group of 25-34years. A greater number (51.7%) of them had up to secondary education and 66.6% worked in the employment category as professionals/administrative work. Most of the women (42.3%) lived in low cost residential areas which imply highly dense settlements, 64.5% of them had no children and 16.8% were HIV positive (Table 1). Twenty-four of the pregnant women out of the 411 who participated in the study were reactive to *Toxoplasma gondii* IgG antibodies, which represented an overall seroprevalence of 5.87% of the total number of pregnant women who attend antenatal clinic at the university teaching hospital. None of these pregnant women were reactive to *Toxoplasma gondii* IgM antibodies.

**Table 1:** Prevalence and Factors Associated with Toxoplasma gondii Infection

			UNIVARITE ANALYSIS			MULTIVARIABLE ANALYSIS		
Characteristics	Response (%) n	Prevalence (%), category total (n)	OR	95% CI	*P*-VALUE	OR	95% CI	*P*-VALUE
SOCIO-DEMOGRAHIC								
AGE IN YEARS								
15-24	(18.7) 77	11.7 (77)	3.11	1.21-7.96	0.02	0.11	0.00-7.41	0.30
25-34	(59.6) 245	4.08 (245)	1.00	1.00	1.00	1.00	1.00	1.00
35-49	(21.7) 89	5.62 (89)	1.40	0.46-4.21	0.55	1.91	0.25-14.7	0.53
LEVEL OF EDUCATION								
Up to secondary*	(51.7) 209	6.22 (209)	1.00	1.00	1.00	0.60	0.10-3.57	0.57
Tertiary	(48.3) 195	4.62 (195)	0.73	0.30-1.75	0.48	1.00	1.00	1.00
MARITAL STATUS								
Married	(88.3) 361	4.99 (361)	1.00	1.00	1.00	1.46	0.01-271	0.89
Never been married	(5.38) 22	18.2 (22)	3.46	1.08-11.1	0.04	10.9	0.06-1955	0.37
Divorced/Widowed	(6.36) 26	7.69 (26)	1.91	0.41-8.79	0.41	1.00	1.00	1.00
EMPLOYED								
In Employment	(62.7) 257							
Unemployed	(37.3) 153							
EMPLOYMENT TYPE								
Farming and construction	(2.7) 7	28.6 (7)	8.97	1.47-54.6	0.02	15.5	0.23-1019	0.20
Professional/Administrative	(63.6) 164	6.90 (87)	1.66	0.54-5.11	0.38	0.71	0.08-6.02	0.75
Trading/other businesses	(33.7) 87	4.27 (164)	1.00	1.00	1.00	1.00	1.00	1.00
INCOME								
Below K3000	(37.6) 94	10.64 (94)	3.13	0.95-10.3	0.06			
Between K3000 and K5000	(43.6) 109	3.67 (109)	1.0	1.00	1.00			
Above K5000	(18.8) 47	2.13 (47)	0.57	0.06-5.25	0.62			
RESIDENCE								
High cost residential area	(18.5) 76	3.95 (76)	0.94	0.23-0.13	0.93	2.84	0.20-39.3	0.44
Medium cost residential area	(39.2) 161	3.73 (161)	2.30	0.64-8.18	0.20	0.40	0.03-5.77	0.50
Low cost residential area	(42.3) 174	8.62 (174)	1.0	1.00	1.00	1.00	1.00	1.00
OBSTETRICAL FACTORS								
GESTATIONAL AGE								
First Trimester	(20.5) 84	3.57 (84)	0.68	0.18-2.53	0.56			
Second Trimester	(32.6) 134	8.21 (134)	1.64	0.68-4.00	0.28			
Third Trimester	(47.0) 192	5.18 (193)	1.0	1.00	1.00			
PARITY								
No child (first pregnancy)	(64.5) 262	4.62 (262)	1.77	0.23-13.9	0.59			
One child	(29.3) 119	9.09 (119)	0.83	0.09-7.80	0.87			
Two and above	(6.16) 25	3.47 (25)	1.0	1.00	1.00			
BEHAVORIAL/LIFESTYLE FACTORS								
CONTACT WITH CATS								
Yes	(10.0) 41	7.32 (41)	1.31	0.37-4.59	0.68	7.81	0.99-61.8	0.05
No	(90.0) 369	5.69 (369)	1.0	1.00	1.00	1.00	1.00	1.00
CONTACT WITH DOGS								
Yes	(22.2) 91	6.59 (91)	1.18	0.45-3.07	0.73			
No	(77.8) 319	5.64 (319)	1.00	1.00	1.00			
EATING MEAT								
Yes	(97.6) 401	5.99 (401)	1.00	1.00	1.00			
No	(2.43) 10	0 (10)	2.65*10-^06^	0	0.99			
LENGTH OF COOKING MEET								
Under 30 min	(12.6) 49	4.08 (49)	0.62	0.14-2.82	0.54			
Within 30 to 60 min	(55.9) 218	6.42 (218)	1.00	1.00	1.00			
Within 60 to 120 min	(18.0) 70	4.29 (70)	0.65	0.18-2.34	0.51			
Over 120 min	(13.6) 53	3.77 (53)	0.57	0.13-2.60	0.47			
EATING CURED MEAT								
Yes	(87.0) 347	5.19 (347)	1.00	1.00	1.00	1.00	0.02-43.6	1.00
No	(13.0) 52	11.5 (52)	2.38	0.90-6.32	0.08	1.00	1.00	1.00
EATING RAW VEGETABLES								
Yes	(81.3) 334	5.69 (334)	1.00	1.00	1.00			
No	(18.7) 77	6.49 (77)	1.15	−4160-0.04	0.79			
DRINKING UNPASTEURIZED MILK								
Yes	(8.76) 36	8.33 (36)	1.53	0.43-5.41	0.51			
No	(91.2) 375	5.60 (375)	1.00	1.00	1.00			
CO-INFECTION								
HIV STATUS								
Positive	(16.8) 69	8.70 (69)	1.69	0.65-4.43	0.28	0.39	0.04-3.47	0.40
Negative	(82.2) 338	5.33 (338)	1.0	1.00	1.00	1.00	1.00	1.00

Associations were calculated with Chi-square, and Fisher’s exact test where appropriate, whereas GLM for binary outcome reporting odds ratios was used for the univariate analysis

* = the category ‘two and above’ was created because the participants who had three to eight (maximum) children were few hence were not very comparable

Variables, ‘eating meat’, parity, ‘length of cooking meat’, ‘drinking unpasteurised milk’ and income were omitted in the final model due to their small numbers

### Determinants of toxoplasmosis

In univariate analysis, age category 15–24 years showed significance to the infection with 3.11 times the likelihood of contracting the infection among this age group as compared to the older groups. Those who had never been married also showed significance, with 3.5 times risk of the disease. Employment type and income, both being characteristic of socio-economic status, also exhibited 8.97 times and 3.13 times the risk of contracting the infection respectively (Table 1).

At multivariable analysis, there was no significant outcome, but for employment type and contact with cats which showed association with the infection with 15.5 times and 7.81 times the risk of the infection in the pregnant women respectively (Table 1).

## Discussion

This study has shown that the overall seroprevalence of *Toxoplasma gondii* infection among pregnant women attending antenatal clinic at UTH is 5.87% and that ‘contact with cats’, ‘employment type’ and socio-economic status are associated with the infection.

Though there are studies with comparatively low seroprevalence of the infection in pregnant women such as in the United Kingdom, 9%, [19], South Africa, 6.4%, [23] and Korea, 3.7%, [19], seroprevalence in this current study is quite low. This is in comparison with results from other studies in the region such as 18.7% in Mozambique [33], 30.9% in Tanzania [22] and 92.5% in Ghana [13]. Globally, 24.1% in Saudi Arabia, [19], 28.3% in Southern Thailand [24] and 17.3% in London [20]. Nonetheless, we find of epidemiological importance the low prevalence and agree with the statements by Kistiah that “A low prevalence means that more previously unexposed people are at risk of acquiring an acute infection, which may cause congenital disease in pregnant women, or which, in reactivation form may ultimately be life-threatening in HIV/AIDS patients” [23] and by Andiappan that, “The seroprevalence may vary in a global view, but the risk of this parasitic infection in human populations, especially in pregnant women, still holds a great interest” [34]. So though the prevalence turned out to be low, it is still of medical importance.

There was no IgM result found in this study. Some studies found IgM antibodies [13, 20, 23], whereas other studies consistent with this study did not find [22, 33, 35]. Most studies report mainly no or few IgM antibodies as compared to IgG. The ELISA method which detects early IgM infections even within the first two weeks of infection seems to be the most appropriate for discovering IgM antibodies [16].

Cats are believed to be the main carriers and transmitters of *T. gondii* infection to man [9] and some studies have established that they are significantly associated with Toxoplasmosis [13, 27]. However, this study did not establish significant association between the infection and ‘contact with cats’ and this is consistent with reports established elsewhere [20, 22, 26, 30, 35]. Albeit, the risk of contracting the infection where there is ‘contact with cats’ in this study was found to be 7.81 times more than where there is no contact with cats. It is important however to mention, that the risk of contracting *Toxoplasma gondii* from cats is as a result of the way or extent of handling the cat litter and not necessarily simple contact with cats [22, 36]. More so, excretion of oocysts (which are infective) by infected cats lasts only few weeks [26]. These we believe are the reasons for the non-significance which was found in this current study as well as the other studies which shared same results.

Eating meat and eating undercooked meat were not significantly associated with Toxoplasmosis in our study. Some studies have reported that eating undercooked meat is a determining factor of Toxoplasmosis [20, 25–27], but the divergent result of this current study turns to concur with other studies [30, 35], which did not find ‘eating undercooked meat’ to be significantly associated with Toxoplasmosis. One reason for this disparity arises from the different types of meat that are investigated into in the various studies [37]: This current study investigated beef, mutton, chevon, chicken, pork and ‘others’ which comprised bush meat or game meat. Again, the varying prevalence of the disease in food producing animals in the various affected regions plays a significant role in the infection in man [14]. The different hygienic conditions under which meat is kept and handled before consumption is also a contributing factor to this [38]. What's more, the definition of undercooked meat may differ among these studies due to different settings such as culture and preference, under which these studies were carried out. This study considered undercooked meat to be meat cooked in less than thirty minutes. This is not a documented universal standard: Undercooked meat in some European countries refers to raw meat which still has blood in it and this is uncommon in our setting. It is unclear however, the criteria other studies used to define undercooked meat. This could be the reason why this variable did not come out as an important determining factor of the infection in this study.

Prevalence of *Toxoplasma gondii* infection among HIV positive pregnant women was higher than that in HIV negative ones in this study. This difference was not statistically significant but is consistent with results from other reported studies [17, 33, 39]. Two studies however report significant association between these two groups [17, 33]. According to one current study, one of the reasons for the non-significance could be as a result of the Anti-Retroviral Therapy (ART) which some of the HIV positive pregnant women were taking [36]. Nonetheless, another study compared the prevalence of the infection in HIV- Toxoplasmosis co-infected individuals on ART and HIV- Toxoplasmosis co-infected individuals not on ART and found no significant association between the two groups [39]. It is however unclear the reason for these results.

‘Farming/ construction under ‘Employment type’ was found to have association with the infection. This category showed 15.5 times the likelihood of contracting the infection as compared to trading/other businesses and professional/administration which had a reduced risk of the infection. Some studies reported significant association with soil related occupation [28], ‘being a labourer’ [34], ‘employed/business women’ as compared to ‘peasants’ [22] as well as weak association with ‘working with animals’ [26]. Some other studies also found ‘occupation’ to have no association with the infection at all [19, 36]. There is an extent of diversity when it comes to ‘occupation’, as different studies investigate different economic activities under this term and others do not specify exactly which activities investigated.

There was increased risk of the infection with increase in age as was reported elsewhere [22, 28, 35] and these studies found this association to be significant. However, consistent with our study’s finding, some studies did not establish significant association between the two [13, 39]. Age appears to be associated with the infection because time of exposure increases as one gets older [35]. Age alone is neither sufficient no necessary for the infection to occur.

Our study further demonstrated no significant association with gestational age as reported in other studies [13, 36] but higher infections were found with the second and third trimesters of both HIV positive and HIV negative pregnant women.

**Fig. 1:**
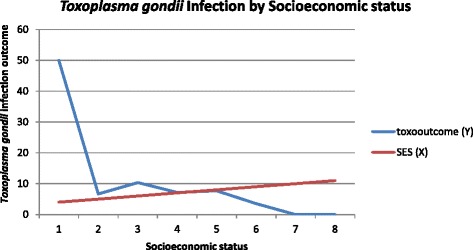
A Graph showing the relationship between *Toxoplasma gondii* infection and Socio-economic status of Study Participants

This study found that there was an association between *Toxoplasma gondii* infection and the socio-economic status of pregnant women. Highest infections where found among pregnant women with low socio-economic status as compared to those with high socio-economic status (see Fig. 1). This was in line with other studies’s findings [21, 34, 40]. This could be due to pregnant women of low socio-economic status being more prone to live and work in highly dense areas with poor sanitary conditions as was realised in this study, lack good education and may lack knowledge on good hygienic practices in general.

### Limitations and strengths of the study

The results of this study cannot be generalised to the entire population in Zambia due to the fact that the study was carried out only in pregnant women in Lusaka. Furthermore, though a negative test result indicates absence of the infection, it does not rule out possibility of exposure to or with the infection. Again, some specimen containing unusually high titer of heterophile antibodies or rheumatoid factor may affect the expected results.

Though these limitations exist, our study is the first of its kind done in pregnant women in UTH and in the country at large and it was carefully and accurately carried out following all necessary precautions. The study population was also a true susceptible population where Toxoplasmosis is concerned, hence the need for the study.

## Conclusion

There is low prevalence of *T. gondii* infection among pregnant women attending antenatal clinic in UTH, Lusaka, Zambia, with a prevalence of 5.87%. Contact with cats, employment type and socio-economic status have association with *Toxoplasma* infection.

The low prevalence in this investigated area could be due to the fact that fewer women own or have contact with cats and also that farming / construction is not an activity greatly engaged in by most women in the urban city, Lusaka.

### Recommendations

Toxoplasmosis screening can be carried out twice (during first and third trimesters) or at least once (during first trimesters) for every pregnant woman, in hospitals. We recommend this because as highlighted early in this study, vertical transmission of the infection during the first trimester is critical and causes severe clinical conditions in the fetus, and third trimester infections have rapid transmission rate of parasites to fetuses, hence causing higher incidence of disease. This when practised, will help save the lives of both mother and child. Health promotion among women of childbearing age is also of immense importance in order to create awareness of the disease in this group and help curb it. In as much as we have given a good picture of the infection among pregnant women in Lusaka, we recommend that more robust studies such as cohort and case–control studies be carried out in the country in order to better ascertain the extent of the condition nationwide.

## Abbreviations

AIDS: Acquired immune deficiency syndrome
ANC: Antenatal care
CI: Confidence interval
CNS: Central nervous system
GLM: Generalised linear model
HIV: Human immunodeficiency virus
IgG: Immunoglobulin G
IgM: Immunoglobulin M
SAG2: Surface antigen 2 gene
*T. gondii*: *Toxoplasma gondii*
UNZA: University of Zambia
US: United States
UTH: University teaching hospital
